# Male dominance linked to size and age, but not to 'good genes' in brown trout (*Salmo trutta*)

**DOI:** 10.1186/1471-2148-7-207

**Published:** 2007-11-01

**Authors:** Alain Jacob, Sébastien Nusslé, Adrian Britschgi, Guillaume Evanno, Rudolf Müller, Claus Wedekind

**Affiliations:** 1 Department of Ecology and Evolution, University of Lausanne, Biophore, 1015 Lausanne, Switzerland; 2 Division of Conservation Biology, University of Bern, Erlachstrasse 9a, 3012 Bern, Switzerland; 3 Eawag: Swiss Federal Institute of Aquatic Science and Technology, Seestrasse 79, 6047 Kastanienbaum, Switzerland

## Abstract

**Background:**

Males that are successful in intra-sexual competition are often assumed to be of superior quality. In the mating system of most salmonid species, intensive dominance fights are common and the winners monopolise most mates and sire most offspring. We drew a random sample of mature male brown trout (*Salmo trutta*) from two wild populations and determined their dominance hierarchy or traits linked to dominance. The fish were then stripped and their sperm was used for *in vitro *fertilisations in two full-factorial breeding designs. We recorded embryo viability until hatching in both experiments, and juvenile survival during 20 months after release into a natural streamlet in the second experiment. Since offspring of brown trout get only genes from their fathers, we used offspring survival as a quality measure to test (i) whether males differ in their genetic quality, and if so, (ii) whether dominance or traits linked to dominance reveal 'good genes'.

**Results:**

We found significant additive genetic variance on embryo survival, i.e. males differed in their genetic quality. Older, heavier and larger males were more successful in intra-sexual selection. However, neither dominance nor dominance indicators like body length, weight or age were significantly linked to genetic quality measured as embryo or juvenile survival.

**Conclusion:**

We found no evidence that females can improve their offspring's genetic viability by mating with large and dominant males. If there still were advantages of mating with dominant males, they may be linked to non-genetic benefits or to genetic advantages that are context dependent and therefore possibly not revealed under our experimental conditions – even if we found significant additive genetic variation for embryo viability under such conditions.

## Background

In mating systems with elaborate male-male competition, the winners usually get most mates and sire most of the offspring [1-10]. Such a skewed male mating success may either be explained by physically limited access of subdominant males to females and/or by female preference for dominant males [11-13]. Females may prefer more dominant and more attractive males because they provide more resources, better parental care [14,15] or better genes for the common offspring [2,16-18]. The latter hypothesis corresponds to the so-called 'good-genes' hypotheses of sexual selection, i.e. variation in genetic quality is then predicted to be linked to male characteristics that influence female mate choice. The problem of how such genetic variation can be maintained under sexual selection is known as the "lek paradox" [19], and a number of possible solutions for this paradox have been offered (reviewed in [20,21]). Although it is still not fully clear how the genetic variation is maintained, there is much evidence in various species that females can gain genetic advantages by preferring males with well-developed attractiveness traits [22]. Whether females gain genetic benefits by mating with dominant males is less clear.

Experimental tests of the 'good-genes' hypotheses of sexual selection usually suffer from at least one of two problems: First, the predicted genetic effects could be confounded with non-genetic effects. This is especially so in species with some form of parental care. Males with more elaborate secondary sexual characters could, for example provide good genes and much paternal care [23,24]. Second, females sometimes adjust their investment in the offspring (e.g. yolk quality in egg) according to their perception of male attractiveness [25,26]. As a consequence of such differential allocation, 'good genes' effects can be confounded with maternal effects. However, some recent *in vitro *fertilization experiments could control for these potential confounding factors. They demonstrate that offspring viability can indeed have a genetic basis that is revealed by potential attractiveness traits [27,28]. In salmonids, not much is known about female preference for attractiveness traits, but males usually fight intensely for access to spawning territories or to females, i.e. intra-sexual selection is often very important [1,8,29-34]. Females seem to generally prefer spawning with dominant males [8,35,36]. Here we test whether male characteristics that are important in intra-sexual selection are also linked to genetic quality.

'Genetic quality' is, in the context of sexual selection, an umbrella term that includes additive ('good genes') and non-additive genetic effects ('compatible genes') on offspring survival [16]. If male dominance is linked to genetic quality and also positively to breeding success, we predict dominance to be linked to the additive genetic variance in fitness, i.e. to variation in 'good genes', since only additive genetic effects can lead to an universally valid order of mate quality while with non-additive genetic effects the order of mate quality would differ for different females [18]. Because embryogenesis is a crucial life-history stage with usually high mortalities [37,38], and male brown trout provide only genes to their offspring, we used embryo survival as a measure of genetic quality, and we used full-factorial breeding designs to separate and compare additive and non-additive genetic effects on embryo survival.

In a first experiment we caught brown trouts (*Salmo trutta forma fario*, Salmonidae), shortly before spawning season and released them into an artificial channel to study intra-sexual selection. We used the outcomes of all male fights to construct a dominance hierarchy and to test whether there are male characteristics that are linked to dominance. We stripped the fish and used their gametes in a 10 males × 8 females full-factorial breeding design (North Carolina II design [39]). The embryos of the resulting 80 families were raised individually under controlled conditions. We then tested whether males differed in their genetic quality, and if so, whether dominance indicators are linked to superior genetic quality. The last two questions were tested again in a second experiment where we determined and analysed embryo and juvenile survival of additional 13 brown trout males from another river. In the first experiment we found that older, heavier and larger males are more dominant in male-male interactions. In both experiments males differ in their genetic quality, but dominant males do not seem to be of superior genetic quality.

## Results

### First experiment

**Figure 1:**
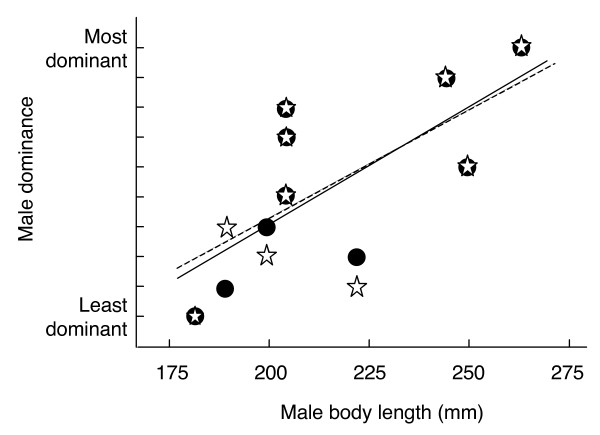
**The effect of male body length on dominance in male-male interactions**. Dominance is given as David's score (circles and non-dashed regression line) and as Clutton-Brock et al.'s index (stars and dashed line). Both scores are based on 198 antagonistic encounters.

Two males were 2 years old, 5 males were 3 years old and 3 males were 4 years old (their body lengths are plotted in Figure 1). Male age, body weight, and body length were all strongly correlated to each other (r always ≥ 0.93, n = 10, p always < 0.0001). Larger males were on average more dominant (Figure 1; with David's score (DS): Spearman's rank order correlation coefficient r_s _= 0.75, p = 0.015; and with Clutton-Brock et al.'s index (CBI): r_s _= 0.68, p = 0.035). We found analogous positive relationship between dominance and male age (DS: r_s _= 0.82, p = 0.006; CBI: r_s _= 0.72, p = 0.02) or male weight (DS: r_s _= 0.73, p = 0.02; CBI: r_s _= 0.64, p = 0.05, n always = 10).

Average embryo survival was 77.9% (± 11.3 s.d.). Offspring of different females differed in their survival as the female effect explained a significant part of the variance in offspring mortality (the model without female effect (*male model*) differed significantly in its goodness of fit from the *reference model*; Table 1). Males also differed significantly in their offspring survival (i.e. the model without the male effect (*female model*) explains significantly less variance in offspring mortality than the *reference model*; Table 1). We found no significant male × female interaction effect on embryo survival (Table 1). The *AIC*s of the different models and the differences between the *AIC*s also indicate that the *reference model *is the most parsimonious one that fits our data best (see Table 1 for details). The fixed temperature effect in the *reference model *was not found to have a significant influence on embryo mortality (Z = -1.718, p = 0.086).

**Table 1:** The Influence of paternal, maternal and paternal × maternal interaction effects on embryo mortality in the 1^st ^experiment.

		Model parameters						Likelihood ratio tests (LRT) with *reference model*		
						
Model	Effect tested	Random	Fixed	Number (k)	*ln L*	*AIC*	Δ*AIC*	χ^2^	d.f.	p
*reference model*		F, M	T	3	-280.19	568.38				
*full model*	Male × Female	F, M, F × M	T	4	-280.19	570.38	2.00	0.00	1	1
*female model*	Male	F	T	2	-283.04	572.07	3.69	5.69	1	0.017
*male model*	Female	M	T	2	-403.65	813.30	244.92	246.92	1	<0.0001

Four logistic mixed effect models are compared to test if male (M), female (F), and male × female interaction (M × F) effects explain a significant part of the variance in embryo mortality (a binary response variable; egg number n = 2028). The random, fixed, and total number (k) of parameters are given for every model. The goodness of fit is given by the logarithm of the approximated likelihood (*ln L*) and the Akaikes information criterion (*AIC*). A measure to compare the quality of fit between two models is the difference of *AICs *(Δ*AIC*) between two models. The *reference model *explains our data best as the more complex *full model *does not significantly improve the qualtiy of fit (see Δ*AIC*; LRT), i.e. the male × female interaction effect did not explain a significant part of the variance in embryo mortality. The table therefore gives the differences in *AICs *between the *reference model *and the other models. Furthermore, likelihood ratio tests (LRT) between the *reference model *and the other models are given to test which parameter significantly improves the goodness of fit.

**Figure 2:**
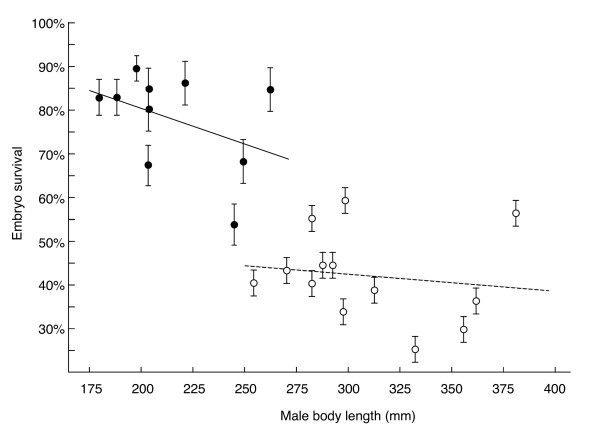
**Embryo survival until hatching (means ± SE) versus male body length**. The survival of visible embryos until hatching, i.e. excluding apparently non-fertilized eggs, for the first experiment (river "Müsche"; filled symbols and non-dashed regression line; Pearson's r = -0.41, n = 10, p = 0.24), and total embryo survival for the second experiment (river "Enziwigger", open symbols and dashed regression line; r = -0.15, n = 13, p = 0.63).

Embryo survival was not positively linked to male dominance (DS: r_s _= -0.47, p = 0.18; CBI: r_s _= -0.48, p = 0.17; n always = 10) and male body length (Figure 2). The 95% confidence interval for the latter correlation is -0.827 < r < 0.295. A power analysis revealed that if the correlation between male length and offspring viability were at the upper limit of our calculated confidence interval, a minimal sample size of 88 males and more than 21,300 experimentally fertilized eggs would be necessary to demonstrate the effect at alpha ≤ 0.05 with our experimental methods and with a statistical power of at least 80%. Since this power analysis is for a possible correlation at the upper extreme of our observed confidence interval, we conclude that there is no or only a very weak positive correlation between male length and offspring survival. There was also no positive link between embryo survival and male age (r = -0.50, n = 10, p = 0.14) or weight (r = -0.40, n = 10, p = 0.25).

### Second experiment

Male age ranged from 3 to 7 years (mean = 4.9 ± 1.2 (s.d.)). Average embryo survival was 42.1% (± 10.0 s.d.). We found again significant sire effects on embryo survival (Table 2), i.e. the males differed in genetic quality. We also found maternal effects on embryo survival, but no significant sire × dam interaction (Table 2). Embryo survival was again not significantly linked to male age (r = -0.19, n = 13, p = 0.54), body weight (r = -0.22, n = 13, p = 0.69), or body length (Figure 2). The correlation coefficient that describes the link between male body length and offspring viability in the second experiment (Figure 2) lies within the 95% confidence interval that we had obtained from the first experiment.

**Table 2:** Variance component analyses on embryo mortality in the 2^nd ^experiment.

	SS	d.f.	F	p	σ^2 ^(% of total)
Sire	1.80	12	2.5	0.01	0.005023 (7.3%)
Dam	0.85	5	2.8	0.02	0.002818 (4.1%)
Sire × dam	3.58	60	1.0	0.53	0 (0%)
Total					0.068507 (100%)

Two-way ANOVA on embryo mortalities observed in the second breeding experiment when 13 males are crossed with 6 females in a full-factorial design and the embryos raised in 3 Petri dishes per sibship. Because the experimental set-up is fully balanced, results are based on EMS (Expected Mean Square). Sire, dam, and sire × dam interaction were random effects in the model. The negative estimate for the variance component of the interaction term is put to zero.

The statistical model in Table 2 explains a significant fraction of the total variance in embryo mortality. Dam effects include direct genetic effects, as well as maternal genetic and maternal environmental effects. Significant sire effects directly reveal variation in genetic quality. Assuming that epistatic genetic variance is of negligible importance, the additive genetic variance can be calculated as four times the sire component of variance [16,39] and explains, in our second experiment, about 29.3% (4 × 0.005023/0.0.068507, see Table 2) of the total phenotypic variance in embryo mortality. The dam × sire effect can be used to estimate the non-additive genetic variance which here represents 0% of the total phenotypic variance in embryo mortality. The difference between the dam and sire component of variance is negative, i.e. the total maternal effect variance seems to be very low in the six females we used here.

**Figure 3:**
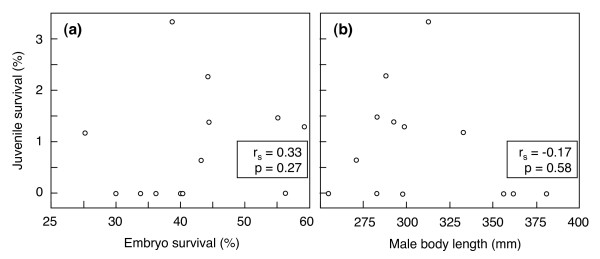
**Juvenile survival in the field (means ± SE) versus embryo survival and male body length**. Juvenile survival during 20 months as determined in the second experiment (total number of juveniles/total number of released hatchlings). The inserts give the Spearman rank order coefficients r_s _and the two-tailed p-values.

We released 2443 hatchlings from this 2^nd ^experiment into the streamlet. Nineteen juveniles could be caught back 20 months later (overall juvenile survival = 0.8%). Juvenile survival was not significantly linked to embryo survival (Figure 3a), and there was no significant positive relationship between juvenile survival and sire body length (Figure 3b), body weight (-r_s _= 0.14, n = 13, p = 0.65), or age (-r_s _= 0.42, n = 13, p = 0.16).

## Discussion

Our first experiment shows that larger, heavier and older males were more dominant in male-male interactions than smaller, lighter and younger ones. This supports findings on other salmonid species where size was a good indicator for dominance status [9,29,40-43]. We have to leave it open whether body length, weight, or age is the better predictor for dominance rank as they were, as expected [44], highly correlated to each other. We used embryo survival until hatching as a main measure of sire 'good genes' because embryogenesis is a critical stage in offspring development (> 50% of offspring mortality normally happens at this stage under natural conditions [37,38]).

We found in both experiments that males differ in their offspring survival. We also tested for female effects on offspring survival. Such latter effects could be explained by differences in genetic quality among the females and/or by differences in egg quality. Variation in egg quality could be linked to female age, condition, and/or life history. Such differences in female investment are expected to be a crucial factor for egg survival in this development stage [45-47]. Accordingly, we found evidence for significant maternal effects on embryo survival in both experiments. Our experimental setup allowed us to control for these female effects. Analogously, males may differ in their sperm quality (e.g. sperm velocity, sperm longevity and spermatocrit), which could influence their fertilisation success [48,49]. We controlled for these potential differences by including only fertilised eggs in our measure of embryo survival in the first experiment. The second experiment was done with males that did not differ significantly in their fertilization ability (Wedekind, unpublished data). Therefore the sire effect that we found is directly linked to differences in male genetic quality and reveals additive genetic variation in embryo viability.

Although males differed in the viability of their embryos and hence in their genetic quality, male dominance or dominance-related characteristics were no indicators of 'good genes'. A power analysis shows that the chance of missing an existing correlation (type II error) is very low. In a second experiment, offspring viability was determined at two stages, as embryo survival in the laboratory and as juvenile survival in the field. In this second experiment we found again differences in male genetic quality but no significant connection between dominance traits and 'good genes'. Hence, we found no support for the hypothesis that dominant males are genetically superior. This seems to be in agreement with previous studies on species with parental care where no link between fathers' dominance and offspring viability was found [50,51], but the relative importance of variation in genetic quality and in parental care remains unclear in these studies.

Because male age, body length, body weight, and dominance are all strongly correlated in brown trout, we expect that older males will have a comparatively high reproductive success simply because they tend to be more dominant. However, according to our results females may not receive genetic benefits from mating with older males, contrary to some predictions from the literature [52-54]. It remains to be tested whether, for males that grow old, an original higher genetic quality is later in life reduced by an accumulation of germ-line mutations [52,55-57]. If so, larger and dominant males may still provide 'good genes' that may, however, only be revealed under certain environmental conditions. The observed embryo survival rates in our laboratory are high compared to more natural conditions [38], especially so in our first experiment, i.e. we incubated the embryos under conditions that may be less challenging than they would usually experience in the wild, giving lower quality embryos a higher survival chance. If so, it is possible that we missed some kinds of sire effects on embryo survival that may be revealed under more challenging conditions.

## Conclusion

Some theory of sexual selection predicts that dominant and older males provide better genes to their offspring than subdominant and younger males. We found that larger, heavier, and older Brown trout male are indeed more dominant in male-male interactions, but females may not improve their offspring's genetic viability by mating with such males. Any advantage of mating with dominant males in brown trout may therefore be linked to a possibly increased fertilization success (but see [58-60]), potential benefits linked to the nest site [3], or to genetic advantages that are more context-dependent and not revealed at our experimental conditions [61] – even if we can demonstrate significant additive genetic variation for embryo viability under such conditions.

## Methods

### First experiment

We caught 10 males by electric fishing in the River Müsche (Kt. Bern, Switzerland) shortly before the breeding season. We then introduced them into an experimental channel (volume = 10 × 0.7 × 0.65 m, with gravel ground and several hiding places) in order to record their dominance behaviour. We recorded the winner and the loser of all antagonistic encounters (n = 198) during 8 observation days over a period of 32 days. The behaviour was recorded with 10 video surveillance cameras (CCD cam 1/3" SONY Super HAD, lens angle 78°, minimum illumination 0.05 Lux, Profiline^®^) linked to a MultiCam GV-1000 System (Ecoline^®^). Antagonistic encounters were defined as interactions between two males that resulted in one male leaving the spot of the interaction, or leaving it first. These interactions usually involved display behaviours, bites, and/or chases. To calculate dominance ranks, we used David's Score (DS) [62,63] and Clutton-Brock et al.'s index (CBI) [64], two methods that take the relative strength of the opponent into account.

After the observation period we recorded male body length, weight and age (determined from yearly growth rings on scale samples). The 10 males and 8 females from the same river were narcotised and the eggs and milt stripped individually into separate containers. The eggs of the females were equally distributed to 10 Petri dishes each. Ten μl of milt of one of the ten males' were added in such a way that all possible sibships (10 × 8 = 80) were produced (full-factorial breeding; [39]). Then, every Petri dish was half filled with water and shaken gently for about 5 seconds. Within the next ten hours all eggs were distributed (one egg per well in 2 ml of water) to 24-well Multiwell Plates (BD Falcon; nontreated polystyrene, flat bottom). The water we used for fertilisation and for incubation was standardized reconstituted water according to the OECD guideline for testing of chemicals [65]. The water volume per developing embryo corresponds to the ratio that [66] had used. Eggs of all 80 combinations (n = 2028 with 23.56 ± 10.24 (mean ± std deviation) eggs per combination) were incubated at one of two incubation temperatures (6.9°C and 8.9°C). Water was not changed during the experiment. Egg viability was measured as the survival of visible embryos until hatching, i.e. we excluded apparently non-fertilized eggs and embryos that died before they were visible under a stereomicroscope (Olympus SZX9).

### Second experiment

Brown trout were collected from their natural spawning place in River Enziwigger (Kt. Luzern, Switzerland) in November by electro-fishing. Thirteen mature males were measured for length and weight, and their age was determined from yearly growth rings on scales sampled below the adipose fin near the lateral line. Their milt was stripped for *in vitro *fertilization of the eggs of six females of the same population in again a full-factorial set-up (North Carolina II design). We used 20 μl milt per 80–100 eggs (see [66], for the detailed methods). The resulting embryos were reared in 3 separate Petri dishes per sibship in 50 ml sand-filtered lake water at 4.7°C (mean number of eggs per Petri dish: 20.4 ± 14.1 s.d.). From day 46 after fertilization on, inviable embryos and hatched larvae were recorded and carefully removed from the Petri dishes with a plastic spoon (in regular intervals of about 10 days each). Water was exchanged twice (at day 76 and day 88 after fertilization). Embryo viability was determined for each Petri dish as the number of hatchlings per total number of eggs.

Alevins were kept in darkness in running water at 7–8°C until all embryos had hatched and most alevins had nearly used up their yolk sac, i.e. until day 131 after fertilization. We then released all fish plus some additional ones (Evanno, unpublished data) into a 600 m long streamlet that is confined by two waterfalls. This structured streamlet has a width of up to half a meter and an average depth of about 10 cm. We removed all trouts by electrofishing and released our fish by carefully distributing them over the full length of the streamlet during a period when water discharge was low and not obviously affecting the larvae. We caught the fish back 20 months later by electrofishing. DNA was extracted from fin clips using the DNeasy Tissue kit (Qiagen) following manufacturer instructions. Eight microsatellite markers were used to determine paternity: *Mst85 *[67], *Mst543AE*, *BS131*, *T3-13 *[68], *AETG1 *[69], *Ssosl417 *[70], *Ssa 171 *[71] and *Str58 *[72]. PCR reactions were performed in 10 μL reaction mixtures containing 2.5 μL of DNA template, 1 × PCR buffer (Qiagen), 1.5–2 mM MgCl2, 0.2 mM dNTPs, 0.5 μM of each primer and 0.25 units of Taq DNA polymerase (Applied Biosystems or Qiagen). PCR profile consisted in 30 iterations of 95°C for 30 s, 50°C (*Mst85*, *BS131*), 55°C (*Ssa 171*, *Ssosl417*, *Str58*), 58°C (*Mst543AE*) or 60°C (*AETG1*, *T3-13*) for 30 s, 72°C for 30 s and a final extension at 72°C for 5 min. PCR products were analyzed with an ABI 3100 automated DNA sequencer (Applied Biosystems) using the Genemapper software (Applied Biosystems). Paternity was established using the CERVUS program [73].

### Statistical analyses

In the first experiment where embryos were raised singly, we analysed embryo mortality as binary response variable with logistic mixed-effect models (every embryo as one independent data point; dead before hatching or hatched). We entered rearing temperature as fixed effect, and parent identity as random male, female, and male × female interaction effects. To test whether male, female and male × female interaction effects explain a significant part of the variance in offspring mortality, we fitted a *"full model" *(including all effects), a *"reference model" *(including temperature, male and female effects only), a *"female model" *(including temperature and female effects only) and a *"male model" *(including temperature and male effects only) and tested if the goodness of fit between models differed. The goodness of fit is given both by the logarithm of the approximated likelihood (*ln L*) and by the Akaikes information criterion (*AIC*)[74]. The latter is based on the *ln L *but punishes for the number of included parameters (k) and is calculated as *AIC*_i _= -2 *ln L*_i _+ 2 k_i_. The *AIC *favours models that have a high goodness of fit with the smallest number of entered parameters. To test if models differ in their goodness of fit, we compared the models with likelihood ratio tests (LRT), calculated as: χ^2 ^= 2(*ln L*_1 _- *ln L*_2_). The degree of freedom is the difference in number of free parameters in the two models. The test statistic is then evaluated under the assumption of asymptotic convergence to a χ^2 ^distribution. A second measure that compares the quality of fit between two models is given as the difference of *AICs *(Δ*AIC*), which is here calculated as Δ_i _*AIC *= *AIC*_i _- *AIC*_Reference Model_. An Δ*AIC *≤ 2 indicates substantial support that the two models do not differ in the quality of fit, values between 4 and 10 indicate some support that they differ in the quality of fit, and Δ*AIC *≥ 10 provide much support that the models differ in their quality of fit [75,76]. Analyses were done with the R software [77] and we used the lme4 package for logistic mixed effect model analyses [78].

Embryo mortality in the second experiment was determined for batches of embryos each. We could therefore calculate a two-way ANOVA with the sire and dam identity and sire × dam interaction as random effects and mortality per Petri dish (square-root arcsin transformed) as response variable. This analysis was done with JMP In statistical package JMP V [79]. Graphical inspection of the juvenile survival data suggested that the assumptions of parametric statistics might be significantly violated and hence non-parametric statistics (Spearman rank order correlation coefficients r_s_) was used. All p-values are two-tailed.

## Authors' contributions

AJ, AB and CW conceived the first experiment and did the experimental breeding. AJ determined the dominance scores. AJ and AB recorded embryo mortality and hatching date. CW and RM conceived the second experiment and did the experimental breeding, CW recorded embryo mortality. RM, CW, and AJ released the hatchlings into the streamlet and caught them back 20 months later. GE did the molecular analyses on the parents and the juveniles of the second experiment. SN, AJ and CW analysed the data. AJ and CW wrote the manuscript. All authors read and approved the final manuscript.
